# A high-quality genome assembly and annotation of the dark-eyed junco *Junco hyemalis*, a recently diversified songbird

**DOI:** 10.1093/g3journal/jkac083

**Published:** 2022-04-11

**Authors:** Guillermo Friis, Joel Vizueta, Ellen D Ketterson, Borja Milá

**Affiliations:** 1 Department of Biodiversity and Evolutionary Biology, National Museum of Natural Sciences, Spanish National Research Council (CSIC), Madrid 28006, Spain; 2 Centre for Social Evolution, University of Copenhaguen, Copenhaguen 1165, Denmark; 3 Department of Biology, Indiana University, Bloomington, IN 47405, USA

**Keywords:** dark-eyed junco, *Junco hyemalis*, genome assembly, Hi-C

## Abstract

The dark-eyed junco (*Junco hyemalis*) is one of the most common passerines of North America, and has served as a model organism in studies related to ecophysiology, behavior, and evolutionary biology for over a century. It is composed of at least 6 distinct, geographically structured forms of recent evolutionary origin, presenting remarkable variation in phenotypic traits, migratory behavior, and habitat. Here, we report a high-quality genome assembly and annotation of the dark-eyed junco generated using a combination of shotgun libraries and proximity ligation Chicago and Dovetail Hi-C libraries. The final assembly is ∼1.03 Gb in size, with 98.3% of the sequence located in 30 full or nearly full chromosome scaffolds, and with a N50/L50 of 71.3 Mb/5 scaffolds. We identified 19,026 functional genes combining gene prediction and similarity approaches, of which 15,967 were associated to GO terms. The genome assembly and the set of annotated genes yielded 95.4% and 96.2% completeness scores, respectively when compared with the BUSCO avian dataset. This new assembly for *J. hyemalis* provides a valuable resource for genome evolution analysis, and for identifying functional genes involved in adaptive processes and speciation.

## Teaser

The dark-eyed junco (*Junco hyemalis*) is one of the most common passerines of North America, and has served as a model organism in different research disciplines for over a century. Here we report a high-quality genome assembly and annotation of the dark-eyed junco generated using a combination of shotgun and proximity ligation libraries. This new assembly for *J. hyemalis* provides a valuable resource for genome evolution analysis, and for identifying functional genes involved in adaptive processes and speciation.

## Introduction 

**Fig. 1.**
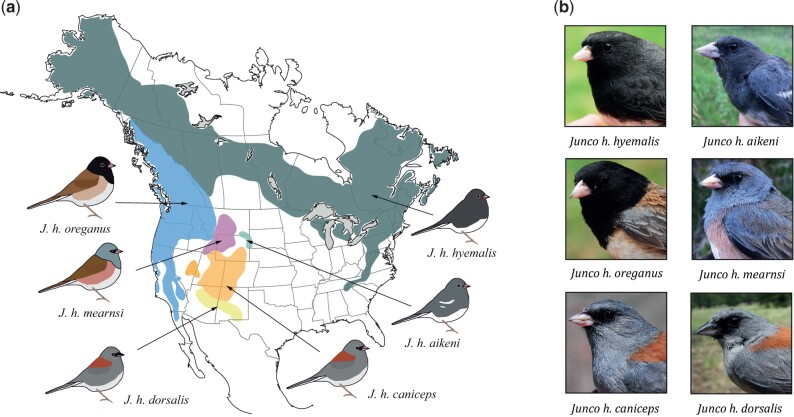
Geographic distribution and phenotypic diversity in the dark-eyed junco. a) Distribution map of the main dark-eyed junco forms. Colored areas correspond to the approximate breeding ranges of each form. b) Photographs of male individuals of the 6 main dark-eyed junco forms (Photos by BM).

The dark-eyed junco (*Junco hyemalis*) is a common and widespread North American passerine that has been the subject of extensive research in multiple scientific disciplines for over 100 years (Miller 1941; Ketterson and Atwell 2016). The species present at least 6 distinct and geographically structured forms, showing marked levels of divergence in plumage coloration, habitat and life-history traits, including the timing of reproduction or migratory behavior (Fig. 1; Supplementary Table 1; Miller 1941; Nolan *et al.* 2002). These forms represent independent evolutionary lineages that radiated after the Last Glacial Maximum circa 18,000 years ago during a northward recolonization of the North American continent (Milá *et al.* 2007; Friis *et al.* 2022). Previous studies based on genome-wide data have documented a unique case of rapid diversification driven by the combined effects of sexual selection, ecological habitat features, and historical demographic factors (Friis *et al.* 2018; Friis and Milá 2020). The dark-eyed junco has also served as a model organism for the study of endocrine, neurological and behavioral aspects of migration, reproductive phenology (e.g. Ketterson and Nolan 1976; Cristol *et al.* 2003; Fudickar *et al.* 2017; Singh *et al.* 2021) and other life-history traits (Ketterson and Nolan 1999); ecophysiology (e.g. Stager *et al.* 2015, 2021); the hormonal basis of phenotypic variation, including traits related to sexual dimorphism, courtship behavior and mate choice (e.g. Ketterson *et al.* 1996, 2005, 2009); differential gene expression associated with pigmentation (Abolins-Abols *et al.* 2018); and phenotypic plasticity and adaptation to urban environments (e.g. Yeh and Price 2004; Atwell *et al.* 2014; Friis *et al.* 2022). For a review, see Ketterson and Atwell (2016). Despite the extensive research conducted on dark-eyed juncos, the molecular basis of the outstanding diversity found in the group remains poorly understood, in part because high-quality, full genome resources accounting for such variability have been sparse.

A plethora of avian species has been fully sequenced and analyzed in the last decade (e.g. Warren *et al.* 2010; Zhang *et al.* 2012; Jarvis *et al.* 2014; Poelstra *et al.* 2014; Liu *et al.* 2019; Louha *et al.* 2020; Dussex *et al.* 2021; Recuerda *et al.* 2021). Recently, the Ten-Thousand Bird Genomes (B10K) consortium released over 300 avian genomes from 92.4% of all avian families, enabling genome-based comparative studies at phylogenetic scales never achieved before (Zhang *et al.* 2014; Feng *et al.* 2020). Yet within birds, the dark-eyed junco complex is of particular interest for the study of evolutionary processes. First, the system (which includes the closely related yellow-eyed junco *Junco phaeonotus* from Mexico and Central America), is composed of recently diversified yet phenotypically differentiated lineages, among which the signals of drift and selection at the molecular level are still recent and detectable. Second, the complex includes forms with broad geographic distributions encompassing heterogeneous habitats across ecological clines, but also spatially discontinuous habitats so that selective and neutral processes of divergence can be assessed in different spatial settings. Third, dark-eyed juncos show large variability in the degree of geographical isolation among phenotypically differentiated forms, from extensive gene flow to total isolation, which along with the first 2 points, makes them a suitable system for studying evolutionary processes related to dispersal, directional selection, and neutral evolution (Milá *et al.* 2006; Friis *et al.* 2018, 2022). Fourth, the dark-eyed juncos present highly divergent secondary sexual traits, enabling comparative analyses to test the role of sexual selection as a driver of genetic divergence (Friis and Milá 2020; Friis *et al.* 2022). And finally, as in most bird species, the dark-eyed junco genome is highly syntenic within the class Aves, relatively small, and structurally simple compared with other amniotes (Gregory 2002), which facilitates the identification of polymorphisms and structural variants.

Here, we report a high-quality, chromosome-level assembly obtained using shotgun and proximity ligation libraries as a resource for genome-based studies on *J. hyemalis* and related bird species. A structural and functional annotation using gene prediction and similarity approaches is also provided. To evaluate the performance of the assembly as a reference for genome-based studies, we conducted a demographic analysis using the pairwise sequentially Markovian coalescent method (PSMC) and the shotgun data generated for the assembly. Shotgun data are also used for the assembly of the mitochondrial genome.

## Materials and methods

### Genome sequencing and assembly

A high-quality genome was produced combining newly generated shotgun reads and sequence data from proximity ligation libraries. Preparation of proximity ligation libraries Chicago and Hi-C, as well as scaffolding with the software pipeline HiRise (Putnam *et al.* 2016; https://dovetailgenomics.com, last accesed on April 10, 2022) was conducted at Dovetail Genomics, LLC. The sequenced sample consisted of muscle tissue obtained from a female *J. hyemalis carolinensis*, collected at Mountain Lake Biological Station in Pembroke, Virginia, USA (37.3751°N, 80.5228°W), currently deposited at the Moore Laboratory of Zoology, Occidental College, Los Angeles, CA, USA (voucher number: MLZ: bird: 69236). Briefly, a de novo draft assembly was first built using shotgun, paired-end libraries (mean insert size ∼350 bp) and the Meraculous pipeline (Chapman *et al*. 2011). For the Chicago and the Dovetail Hi-C library preparation, chromatin was fixed with formaldehyde. Fixed chromatin was then digested with DpnII and free blunt ends were ligated. Crosslinks were reversed and the DNA was purified from protein. Resulting nucleic material was then sheared to ∼350 bp mean fragment size and sequencing libraries were generated using NEBNext Ultra enzymes and Illumina-compatible adapters. Sequencing of the libraries was carried out on an Illumina HiSeq X platform. The shotgun reads, Chicago library reads, and Dovetail Hi-C library reads were then used as input data for HiRise, a software pipeline designed specifically for using proximity ligation data to scaffold genome assemblies (Supplementary Fig. 1, Putnam *et al.* 2016). More details on genome sequencing and assembly methods are available in the Supplementary Information.

A circularized assembly of the mitochondrial genome was generated using NOVOplasty2.7.2 (Dierckxsens *et al.* 2017) and the shotgun data. The NADH dehydrogenase subunit 2 (ND2) mitochondrial gene sequenced for a previous study (Friis *et al.* 2022) and available at NCBI (GenBank accession no. KX461682.1) was used for the input seed sequence.

### Identification of repetitive regions

We first created a repeat library for the junco genome by modeling ab initio repeats using Repeat Modeler 1.0.11 (Smit and Hubley 2019) in scaffolds longer than 100 Kb with default options. The resulting repeat library was merged with known bird repeat libraries from the RepBase database (RepBase-20181026) (Bao *et al.* 2015), Dfam_Consensus-20181026 and repeats from the zebra finch. Then, we used Repeat Masker 4.0.7 (Smit *et al.* 2015) to identify and mask repeat regions in the whole-genome assembly and classified the repeat distribution by chromosome.

### Gene prediction and functional annotation

Gene prediction was conducted using BRAKER v2.1.5 (Hoff *et al.* 2019) and GeMoMa v1.7.1 (Keilwagen *et al.* 2019). We used the repeat soft-masked genome assembly and we first trained Augustus with the conserved orthologous genes from BUSCO Aves_odb10 as proteins from short evolutionary distance (Gremme *et al.* 2005; Stanke *et al.* 2006; see Fig. 3b from Hoff *et al.* 2019). The predicted gene models obtained with Augustus were combined with homology-based annotations using the zebra finch (GCF_008822105.2; Warren *et al.* 2010) (GCF_008822105.2; Warren *et al.* 2010) and chicken (GCF_000002315.6; International Chicken Genome Sequencing Consortium 2004) annotated genes with the GeMoMa pipeline, obtaining the final reported gene models. We then applied a similarity-based search approach to conduct the functional annotation of the junco predicted proteins. We first used BLASTP against the UniProt SwissProt database and the annotated proteins from the zebra finch genome (Warren *et al.* 2010; UniProt Consortium 2019) (*E*-value 10^−5^). We only considered as positives those hits covering at least 2/3 of the query sequence length or 80% of the total subject sequence. We also used InterProScan v5.31 (Jones *et al.* 2014) to identify specific protein-domain signatures in the predicted genes. The functional annotation, including gene ontology (GO) terms, was integrated from all searches providing a curated set of junco coding genes (Supplementary Fig. 2). In addition, a visual summary of recovered GO categories for annotated genes was generated with WEGO 2.0 (Ye *et al.* 2018). We also used GenomeTools (Gremme *et al.* 2013) to calculate the number and mean length of genes, exons, introns, and CDS (coding sequence) from the annotation file in general feature format (GFF).

### Gene completeness assessment and genome synteny

We assessed gene completeness in the genome assembly and the gene annotation using BUSCO (Benchmarking Universal Single-Copy Orthologs) v4.0.5 (–auto-lineage-euk option; Waterhouse *et al.* 2018). BUSCO evaluations were conducted using the 255 and 8,338 single-copy orthologous genes in Eukaryota_odb10 and Aves_odb10 datasets, respectively. In addition, we used MUMmer (Delcher *et al.* 2003) to explore synteny with the zebra finch (*Taeniopygia guttata*) genome bTaeGut2.pri.v2 available at NCBI under the accession GCA_009859065.2.

### Historic changes in effective population size

The pairwise sequentially Markovian coalescent (PSMC) (Li and Durbin 2011) was used to model historic changes in the effective population size of the dark-eyed junco. The shotgun data produced was mapped with bwa-mem (Li and Durbin 2009) against the fully assembled genome and a whole-genome diploid consensus sequence was generated using SAMtools v0.1.19 (Li *et al.* 2009) and bcftools (Danecek and McCarthy 2017). The default settings of PSMC were adopted, with a generation time of 1.5 years and a mutation rate of 4.6 × 10^−8^ per site per generation (Nam *et al.* 2010; Smeds *et al.* 2016). Confidence intervals were estimated based on bootstrapping (Li and Durbin 2011).

## Results and discussion

### Sequencing and genome assembly

We sequenced and assembled a reference genome of the dark-eyed junco. Shotgun library produced 465 million read pairs (2 × 150 bp). Chicago and Dovetail Hi-C libraries produced 218 million and 121 million read pairs (2 × 151 bp), respectively. Overall, 121 Gb were generated. Genome scaffolding with HiRise yielded an assembly of 4,684 scaffolds and 1.03 Gb, with a sequence coverage of 117x; an L50/N50 equal to 5 scaffolds/71.3 Mb and an L90/N90 of 19 scaffolds/14.1 Mb; and a relatively low number of ambiguous bases (i.e. N) inserted in the genome (3.13%; Table 1).

**Table 1.** Summary statistics for the genome assembly of *Junco hyemalis.*

Genome assembly	
Total length (bp)	1,031,523,571
Number of scaffolds	4,684
N50/L50	71,317,294 bp/5 scaffolds
N90/L90	14,099,349 bp/19 scaffolds
Chromosome scale	1,013,712,310 bp/30 scaffolds
Longest scaffold (bp)	152,011,357
Missingness	3.13%
GC content	41.85%
BUSCO Eukaryota database	C: 92.9% [S: 92.5%, D: 0.4%], F: 3.9%, M: 3.2%, N: 255
BUSCO Aves database	C: 95.4% [S: 95.2%, D: 0.2%], F: 1.6%, M: 3.0%, N: 8,338

CDS indicates protein-coding sequences. BUSCO parameters are C: Complete BUSCO; S: Complete and single-copy BUSCOs; D: Complete and duplicated BUSCOs; F: Fragmented BUSCOs; M: Missing BUSCOs; and N: Total BUSCO groups searched

**Fig. 2.**
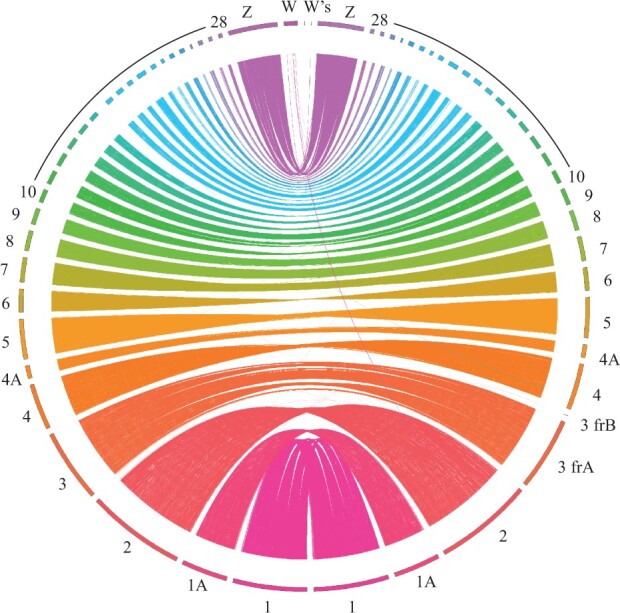
Circos plot showing synteny patterns between the zebra finch (left hemisphere) and the dark-eyed junco (right hemisphere) genome assemblies. Chromosome 3 is represented by 2 scaffolds (frA and frB). Only the 2 largest scaffolds of the 293 that mapped against the W chromosome are shown.

The dark-eyed junco genome also showed high levels of synteny with the zebra finch *Taeniopygia guttata* (Fig. 2). The 30 longest scaffolds obtained based on the proximity ligation mate-paired libraries, ranging from 152.01 to 2.67 Mb (median = 18.90 Mb; Supplementary Fig. 4) and accounting for 98.27% of the whole assembled genome, were identified as full or nearly full chromosomes when compared to the zebra finch (Fig. 2). Both sexual chromosomes were successfully mapped, yet the chromosome W was highly fragmented (293 fragments), possibly due to difficulties assembling highly repetitive genomic regions (Dovetail, pers. comm.). In addition, a circularized assembly of the mitochondrial genome was 16,894 bp long with a 46.6% GC content.

The integrity assessment of the *J. hyemalis* genome retrieved 92.9% and 95.4% of the tested BUSCO groups for the eukaryotes and aves databases, respectively, evidencing the completeness of our assembly (Table 1).

### Genome annotation

We identified 19,026 protein-coding genes, from which 15,967 were associated to GO terms. The average gene length was 15.4 Kb, with a mean of 9.86 exons per gene. BUSCO integrity analysis reported 89.0% of recovered complete BUSCOs for the eukaryota database, and 96.2% in the case of Aves. Quantitatively, our genome annotation identified a considerably higher number of genes, coding sequences, exons and introns when compared with other passerines (i.e. *Fringilla coelebs, Melospiza melodia, Taeniopygia guttata, Ficedula albicollis, Manacus vittelinus*, and *Geospiza fortis*), while averaged lengths for these elements remained similar to other species annotations (Table 2). We also found that a total of 5.77% (59.5 Mb) of the *J. hyemalis* assembly consisted of repetitive elements, a value within the range expected for birds which is at 4%–10% of the genome (Zhang *et al.* 2014). Of the total of repetitive elements, the greatest proportions corresponded to long interspersed nuclear elements (LINEs, 52.51%) and to long terminal repeats (LTRs, 36.57%; Supplementary Table 2). In a WEGO analysis, the most represented GO terms where those associated to “cell,” “cell parts,” and “membrane part” in the cellular component category; “binding” in molecular functions; and “cellular processes” in the category of biological processes (Supplementary Fig. 4).

**Table 2.** Summary statistics for the genome annotation of *Junco hyemalis* compared with other similarly sized avian species (*Fringilla coelebs, Melospiza melodia, Taeniopygia guttata, Ficedula albicollis, Manacus vitellinus*, and *Geospiza fortis*), modified from Recuerda *et al.* (2021).

Genome annotation	*J. hyemalis*	*F. coelebs*	*M. melodia*	*T. guttata*	*F. allbicollis*	*M. vittelinus*	*G. fortis*
Number of genes	19,026	17,703	15,086	17,561	16,763	18,976	14,399
Average gene length (bp)	15,402	15,818	14,457	26,458	31,394	27,847	30,164
Number of CDS	23,245	17,703	15,086	17,561	16,763	18,976	14,399
Average CDS length (bp)	1,647	1,679	1,325	1,677	1,942	1,929	1,766
Number of exons	229,210	221,872	131,940	171,767	189,043	190,390	164,721
Average exon length (bp)	167	165	153	255	253	264	195
Average number of exons/gene	9.86	10.16	8.67	10.25	12.22	11.51	11.41
Number of introns	205,965	200,041	116,724	153,909	171,236	171,089	149,563
Average intron length (bp)	1,945	1,902	1,695	2,930	3,257	3,294	2,813
**BUSCO Eukaryota database:** C: 89.0% [S: 85.1%, D: 3.9%], F: 5.1%, M: 5.9%, N: 255							
**BUSCO Aves database:** C: 96.2% [S: 83.7%, D: 12.5%], F: 1.5%, M: 2.3%, N: 8,338							

BUSCO parameters are C: Complete BUSCO; S: Complete and single-copy BUSCOs; D: Complete and duplicated BUSCOs; F: Fragmented BUSCOs; M: Missing BUSCOs; and N: Total BUSCO groups searched. CDS indicates protein-coding sequences.

### Historic changes in effective population size

**Fig. 3.**
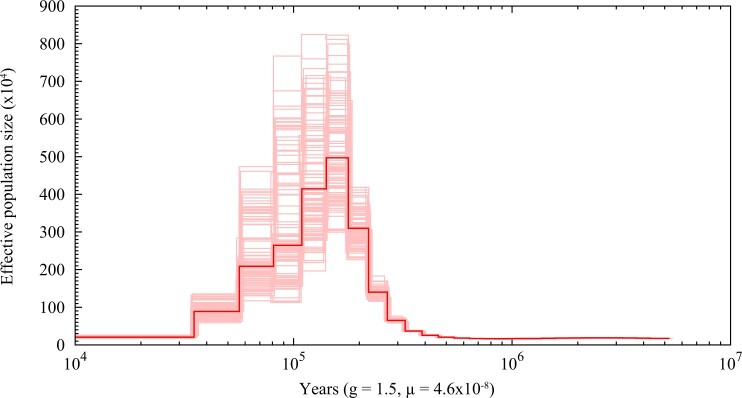
Changes in the ancestral effective population size of the dark-eyed junco. The dark red line represents the original effective population size through time, and light red lines represent 100 bootstrap estimates. The indices g and µ denote the generation time and the mutation rate, respectively.

A PSMC analysis for the dark-eyed junco revealed an ancestral demographic expansion that started 500K years ago approximately. The trend was sustained till circa 100K years ago, followed by a decrease in the effective population size. This decrease roughly overlaps with the onset of the last glaciation period (Richmond and Fullerton 1986), which likely caused a demographic decline of *Junco* populations in the North American continent (Fig. 3).

### Conclusion

We report here a high-quality assembly for the *J.* *hyemalis* genome along with a comprehensive functional annotation, a valuable resource to address a range of biological questions using genomic approaches. The combination of shotgun and proximity ligation libraries yielded an assembly 1.03 Gb long, with 98.3% of the sequence located in 30 full or nearly full chromosome scaffolds. The genome is highly contiguous and complete, enabling its use as a reference for variant calling and for the identification of candidate genes potentially involved in phenotypic variation. Improved scaffolding also enables the identification of regions putatively under selection, including structural variants such as chromosome rearrangements, repetitive elements or copy number variation, all relevant for investigating questions related to evolutionary biology and molecular ecology in the dark-eyed junco system and related species.

## Data availability

Genome assembly, mitochondrial genome, genome annotation and related supporting resources have been deposited at DRYAD (doi: https://doi.org/10.5061/dryad.c59zw3r87). The genome assembly, including the raw shotgun sequencing data, Chicago and Hi-C libraries have been deposited at NCBI under accession QZWM00000000.2; BioProject PRJNA493001; BioSample: SAMN10120167.

Supplemental material is available at *G3* online.

## Supplementary Material

jkac083_Supplementary_Data
