# Supplementary material for: A high-quality genome assembly and annotation of the dark-eyed junco Junco hyemalis, a recently diversified songbird
Source: G3 (Bethesda). 2022 Apr 11;12(6):jkac083. doi: 10.1093/g3journal/jkac083 (PMC9157146; doi:10.1093/g3journal/jkac083)
Supplement: jkac083_Supplementary_Data [file jkac083_supplementary_data.docx]

**Supplementary Information:** **A high-quality genome assembly and annotation of the dark-eyed junco *Junco hyemalis,* a recently diversified songbird**

Guillermo Friis, Joel Vizueta, Ellen D. Ketterson, Borja Milá

**Sequencing and assembly of the *Junco hyemalis* genome**

*Chicago library preparation and sequencing*

A Chicago^TM^ library was prepared as described previously (Putnam et al, 2016). Briefly, ~500ng of HMW gDNA (mean fragment length = 80 Kb) was reconstituted into chromatin in vitro and fixed with formaldehyde. Fixed chromatin was digested with DpnII, the 5’ overhangs filled in with biotinylated nucleotides, and then free blunt ends were ligated. After ligation, crosslinks were reversed, and the DNA purified from protein. Purified DNA was treated to remove biotin that was not internal to ligated fragments. The DNA was then sheared to ~350 bp mean fragment size and sequencing libraries were generated using NEBNext Ultra enzymes and Illumina-compatible adapters. Biotin-containing fragments were isolated using streptavidin beads before PCR enrichment of each library. The libraries were sequenced on an Illumina HiSeq X platform to produce 218 million 2x151bp paired end reads, which provided 233.08 x physical coverage of the genome (1-50kb pairs).

*Dovetail Hi-C^TM^ library preparation and sequencing*

A Dovetail HiC^TM^ library was prepared in a similar manner as described previously (Erez Lieberman-Aiden et al., 2009). Briefly, for each library, chromatin was fixed in place with formaldehyde in the nucleus and then extracted Fixed chromatin was digested with DpnII, the 5’ overhangs filled in with biotinylated nucleotides, and then free blunt ends were ligated. After ligation, crosslinks were reversed, and the DNA purified from protein. Purified DNA was treated to remove biotin that was not internal to ligated fragments. The DNA was then sheared to ~350 bp mean fragment size and sequencing libraries were generated using NEBNext Ultra enzymes and Illumina-compatible adapters. Biotin-containing fragments were isolated using streptavidin beads before PCR enrichment of each library. The libraries were sequenced on an Illumina HiSeq X platform to produce 121 million 2x151 bp paired end reads, which provided 18,311.10 x physical coverage of the genome (1-50kb pairs).

De novo *assembly of the dark-eyed junco genome*

A *de novo* assembly was constructed using a combination of paired end (mean insert size ~350 bp) using Meraculous (v. 2.2.2.5 diploid_mode 1) (Chapman et al, 2011) with a kmer size of 55. The input data consisted of 465.3 million read pairs sequenced from paired-end libraries (totaling 140 Gb). Reads were trimmed for quality, sequencing adapters, and mate pair adapters using Trimmomatic (Bolger et al 2015).

*Scaffolding the assembly with HiRise*

The input de novo assembly, shotgun reads, Chicago library reads, and Dovetail Hi-C library reads were used as input data for HiRise, a software pipeline designed specifically for using proximity ligation data to scaffold genome assemblies (Putnam et al, 2016). An iterative analysis was conducted. First, shotgun and Chicago library sequences were aligned to *de novo* genome assembly. The aligning was conducted using a modified SNAP read mapper (http://snap.cs.berkeley.edu). The separations of Chicago read pairs mapped within draft scaffolds were analyzed by HiRise to produce a likelihood model for genomic distance between read pairs, and the model was used to identify and break putative miss-joins, to score prospective joins, and make joins above a threshold. After aligning and scaffolding Chicago data, Dovetail Hi-C library sequences were aligned and scaffolded following the same method. After scaffolding, shotgun sequences were used to close gaps between contigs. (Figure S1).

**Table S1.** Taxonomy of dark-eyed junco forms based on Miller (1941) and Nolan et al. (2002).

| **Country** | **Species** | **Forms** | **Common name** | **Code** |
| --- | --- | --- | --- | --- |
| USA and Canada | *J. hyemalis* | *hyemalis*  *cismontanus*  *carolinensis* | Slate-colored junco | SCJU |
|  |  | *aikeni* | White-winged junco | WWJU |
|  |  | *caniceps* | Gray-headed junco | GHJU |
|  |  | *dorsalis* | Red-backed junco | RBJU |
|  |  | *mearnsi* | Pink-sided junco | PSJU |
|  |  | *oreganus*  *shufeldti*  *montanus*  *pinosus*  *thurberi*  *pontilis*  *townsendi* | Oregon junco | ORJU |
| Mexico |  |  |  |  |

**Table S2.** Results of the repetitive element annotation conducted with RepeatModeler v2.0.1 and RepeatMasker 4.0.9 (Flynn et al. 2019; Smit et al. 2015). Abbreviated types of elements correspond to short and long interspersed nuclear elements (SINEs and LINEs, respectively) and long terminal repeats (LTRs).

| **Element type** | **Number of elements** | **% of total RE** | **% of the genome** | **Length (bp)** |
| --- | --- | --- | --- | --- |
| SINEs | 1,617 | 0.17% | 0.01% | 114,413 |
| LINEs | 116,487 | 52.51% | 3.03% | 31,260,825 |
| LTR elements | 63,886 | 36.57% | 2.11% | 21,796,149 |
| DNA elements | 7,501 | 2.08% | 0.12% | 1,242,706 |
| Unclassified | 16,785 | 8.49% | 0.49% | 5,065,665 |
| Total interspersed repeats | 206,276 |  | 5.77% | 59,479,758 |

**Fig. S1.** HiRise scaffolding workflow using *de novo* genome along with Chicago and Hi-C data for a final, high-quality assembly. Modified from Dovetail Genomics, LLC sequencing report.


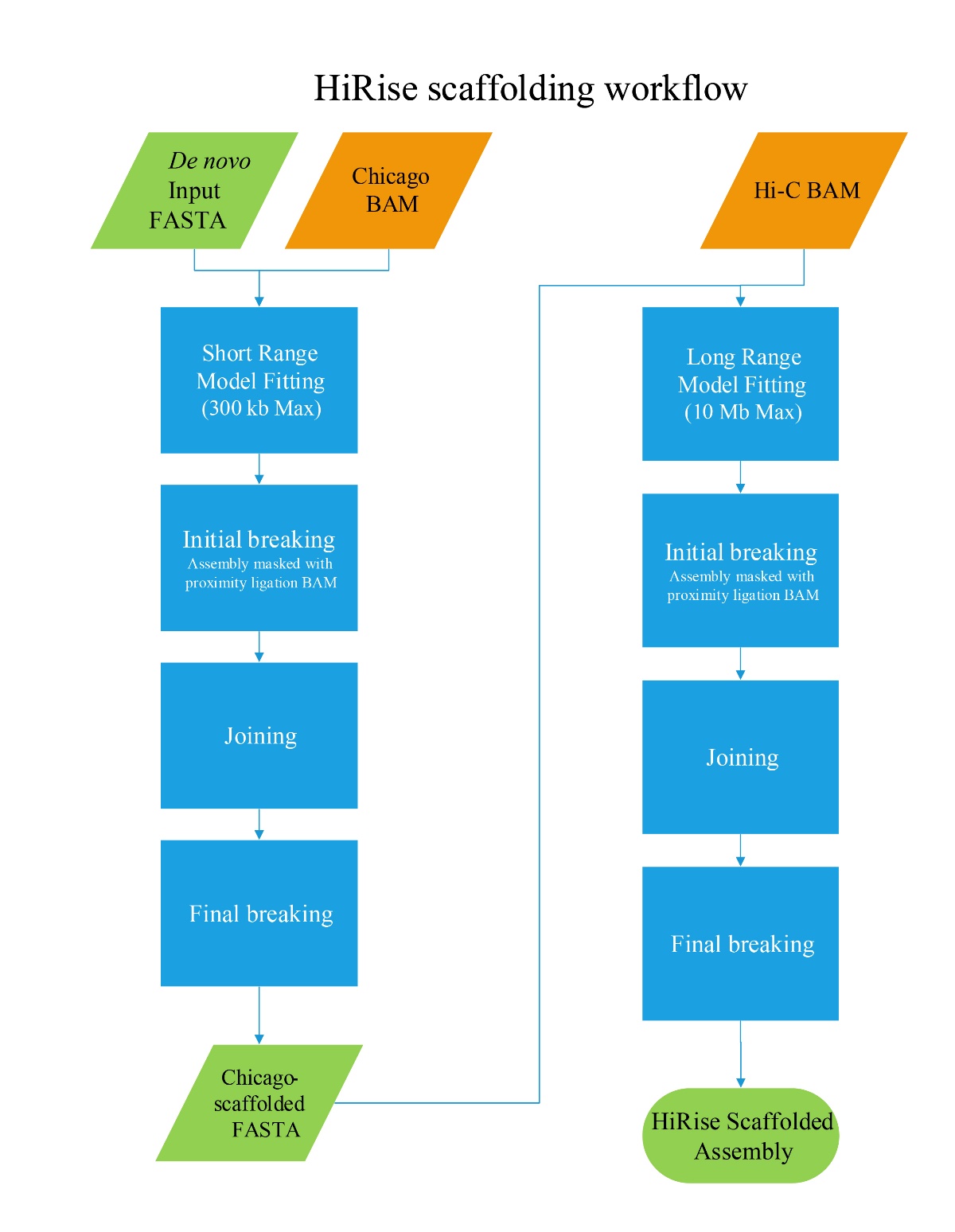


**Fig. S2.** Annotation workflow. Input/output files are showed in orange boxes, while intermediate steps are showed in blue. The steps corresponding to the predictive and functional annotation are showed in light yellow. File formats are reported in parentheses when applicable. Main output files (those reported in the annotation) are showed in bold.


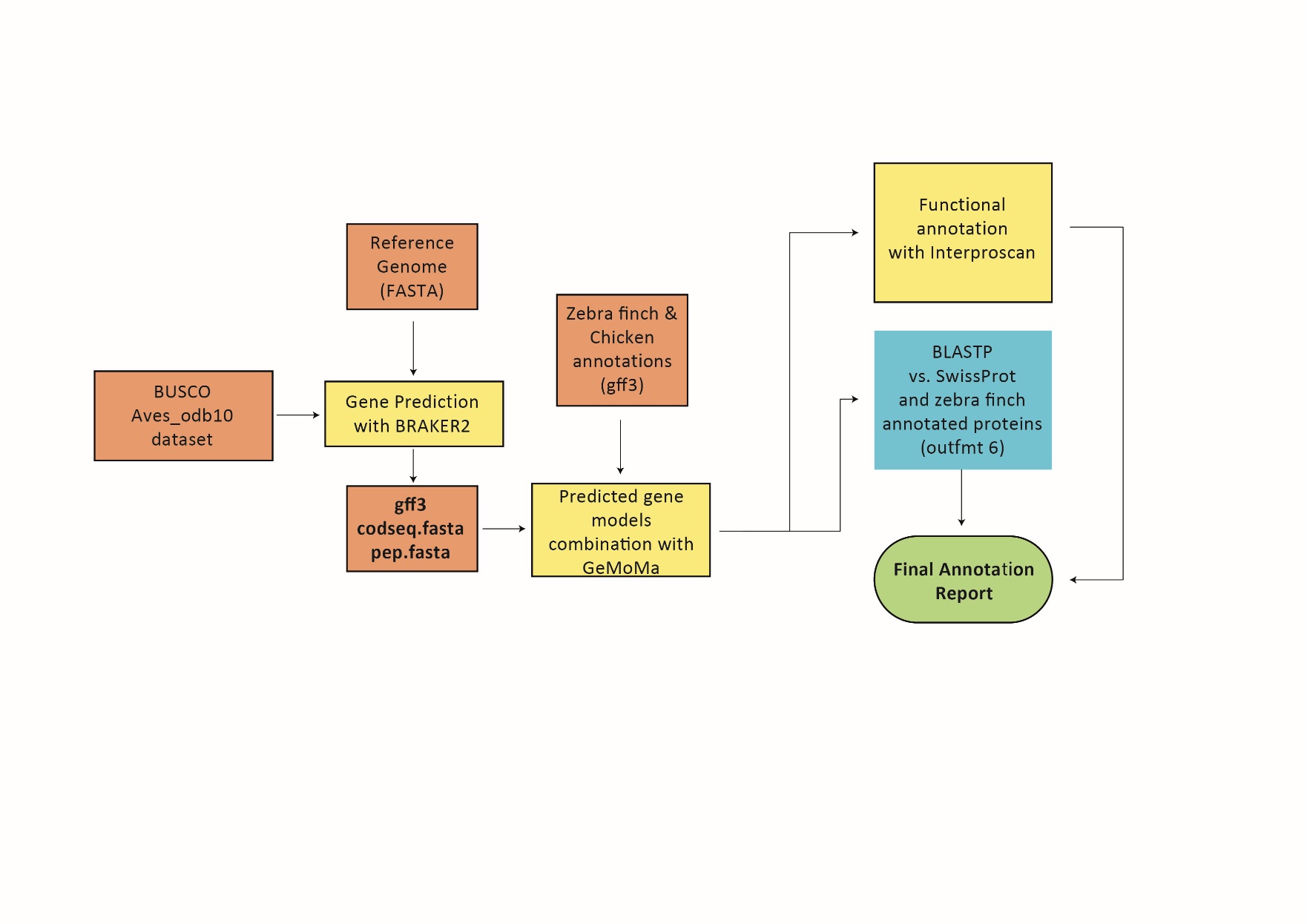


**Fig. S3.** Link density histogram for the dark-eyed junco assembly based on proximity ligation libraries. The horizontal and vertical axes give the mapping positions of the first and second read in the read pair respectively, grouped into bins. The color of each square gives the number of read pairs within that bin. Scaffolds shorter than 1 Mb are excluded. Provided by Dovetail Genomics, LLC.


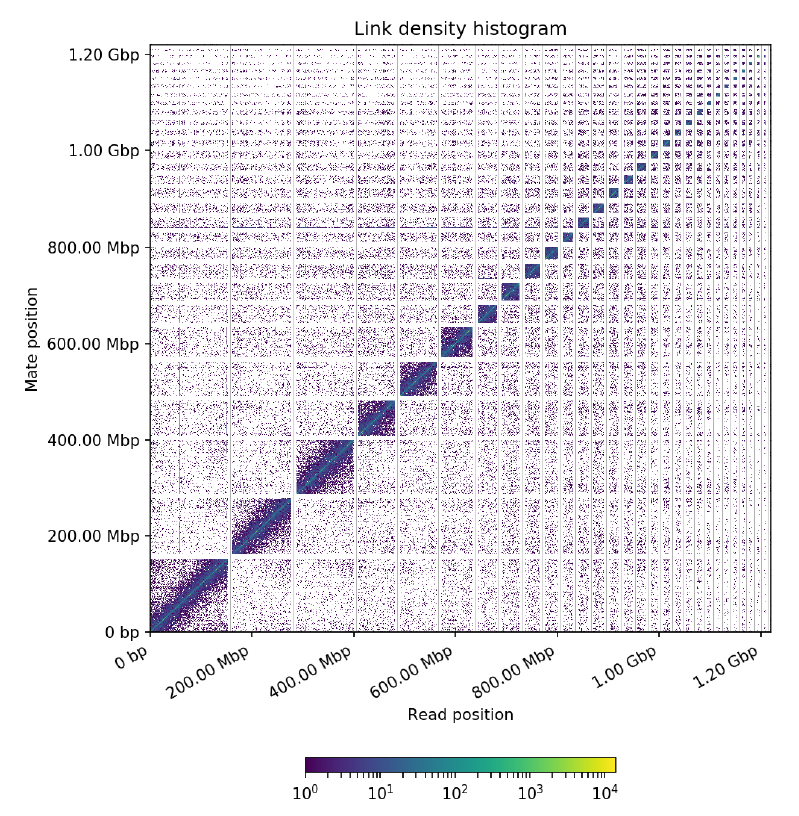


**Fig. S4.** WEGO plot of the GO terms associated with 15,697 functionally annotated genes of the *Junco hyemalis* genome.


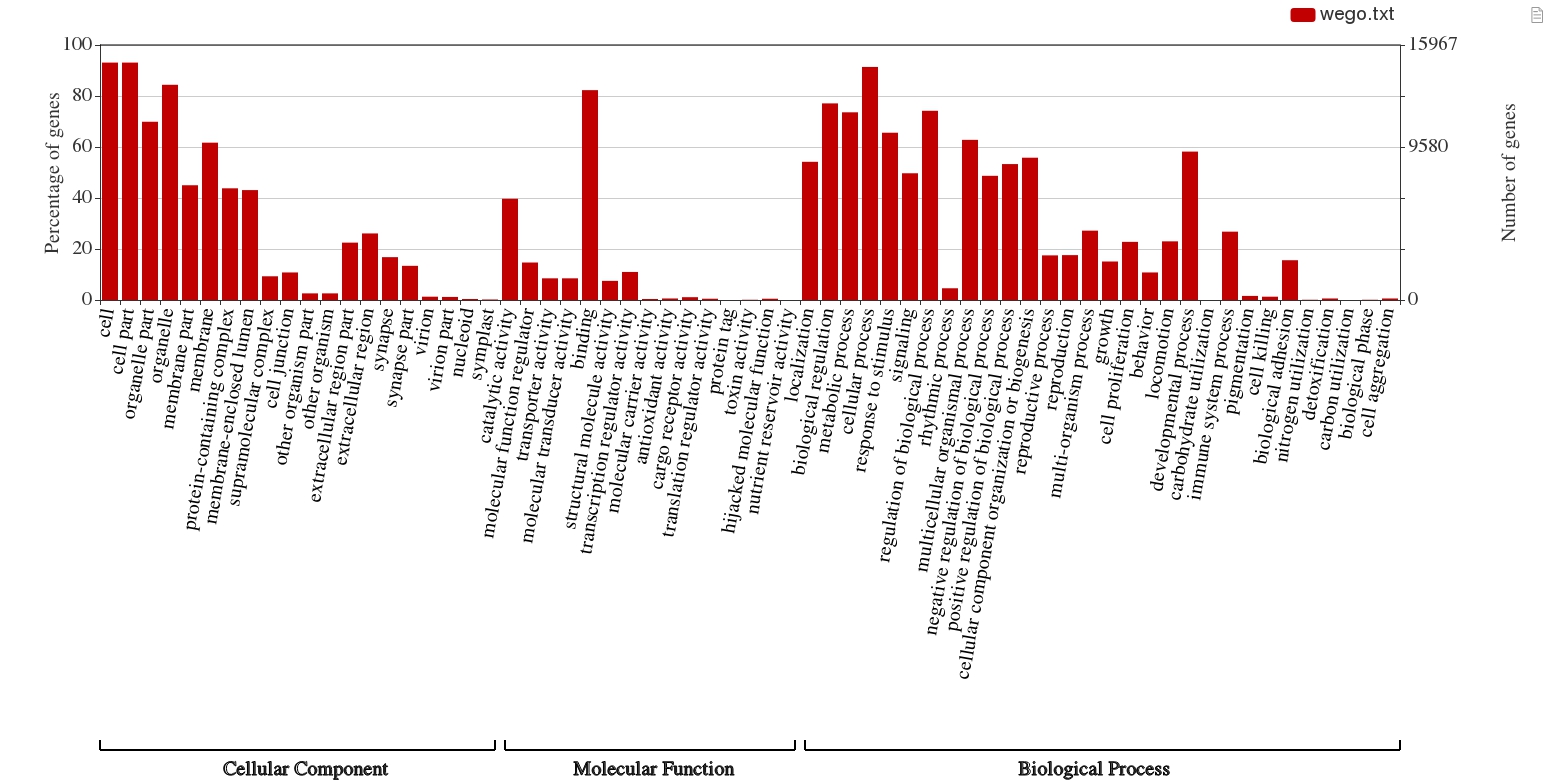


**References**

Flynn, J.M., R. Hubley, C. Goubert, J. Rosen, A.G. Clark *et al.*, 2019 RepeatModeler2: automated genomic discovery of transposable element families. *bioRxiv*:856591.

Miller, A., 1941 Speciation in the avian genus *Junco*. *University of California Publications in Zoology* 44 (3):173-434.

Nolan, V.J., E.D. Ketterson, D.A. Cristol, C.M. Rogers, E.D. Clotfelter *et al.*, 2002 Dark-eyed Junco (*Junco hyemalis*) in *The Birds of North America*, edited by A. Poole and F. Gill. The Birds of North America, Inc., Philadelphia, Pennsylvania.

Smit, A., R. Hubley, and P. Green, 2015 RepeatMasker Open-4.0. 2013–2015.
